# Analysis of the *PRA1* Genes in Cotton Identifies the Role of *GhPRA1.B1-1A* in *Verticillium dahliae* Resistance

**DOI:** 10.3390/genes13050765

**Published:** 2022-04-26

**Authors:** Na Wu, Wen-Jie Li, Chen Chen, Yan-Peng Zhao, Yu-Xia Hou

**Affiliations:** 1 Zhengzhou Research Base, State Key Laboratory of Cotton Biology, School of Agricultural Sciences, Zhengzhou University, Zhengzhou 450001, China; w15093389925@163.com (N.W.); 13781949770@163.com (W.-J.L.); c18337103690@163.com (C.C.); 2 State Key Laboratory of Cotton Biology, Institute of Cotton Research, Chinese Academy of Agricultural Sciences, Anyang 455000, China; 3 College of Science, China Agricultural University, Beijing 100193, China

**Keywords:** *GhPRA1.B1-1A*, *PRA1*, upland cotton, *Verticillium dahliae*, Verticillium wilt

## Abstract

Verticillium wilt in cotton (*Gossypium hirsutum*) is primarily caused by *Verticillium dahliae*. Previous data suggest that prenylated RAB acceptors (PRAs) play essential roles in environmental plant adaptation, although the potential roles of PRA1 in cotton are unclear. Therefore, in this study, PRA1 family members were identified in *G. hirsutum*, and their roles in biotic and abiotic stresses were analyzed. Thirty-seven GhPRA1 family members were identified in upland cotton, which were divided into eight groups. Gene structure and domain analyses revealed that the sequences of GhPRA1 members in each group were highly conserved. Many environmental stress-related and hormone-response *cis*-acting elements were identified in the *GhPRA1* promoter regions, indicating that they may respond to biotic and abiotic stresses. Expression analysis revealed that *GhPRA1* members were widely expressed in upland cotton. The *GhPRA1* genes responded to abiotic stress: drought, cold, salt, and heat stress. *GhPRA1.B1-1A* expression increased after *V. dahliae* infection. Furthermore, the functional role of *GhPRA1.B1-1A* was confirmed by overexpression in *Arabidopsis thaliana*, which enhanced the resistance to *V. dahliae.* In contrast, *V. dahliae* resistance was significantly weakened via virus-induced gene silencing of *GhPRA1.B1-1A* in upland cotton. Simultaneously, reactive oxygen species accumulation; the H_2_O_2_, salicylic acid, and jasmonic acid contents; and callose deposition were significantly decreased in cotton plants with *GhPRA1.B1-1A* silencing. These findings contribute to a better understanding of the biological roles of *GhPRA1* proteins and provide candidate genes for cotton breeders for breeding *V. dahliae*-resistant cultivars.

## 1. Introduction

Cotton (*Gossypium* spp.) is the major crop for natural fiber quality and production and is an important source of edible oil and feed for livestock [1]. Upland cotton (*G. hirsutum*) is the most widely grown cotton species, accounting for approximately 90% of cotton production [2].

Verticillium wilt (V. wilt) is one of the most serious diseases affecting fiber production. V. wilt in cotton is primarily caused by *Verticillium dahliae*, and it can survive in the soil for long periods of time in the form of microsclerotia [3]. The leaves of cotton plants with V. wilt show symptoms of wilting and yellowing, browning occurs in the vascular bundles, and, in serious cases, the whole plant will wither and die. Blockage theory and toxin theory have been used to explain the pathogenesis of V. wilt [4]. According to blockage theory, when *V. dahliae* infects cotton roots, the hyphae pass through the roots of plants and colonize the vascular bundles [5]. The accumulation of mycelia blocks the vessels of xylem tissues, which affects the transportation of water and nutrients in plants. The toxin hypothesis holds that leaf necrosis occurs owing to toxins produced during fungal infection, which ultimately leads to the death of plants [6]. At present, barely effective strategies have been developed to protect cotton plants from *V. dahliae* infection. Thus, understanding the genetic basis of susceptibility to *V. dahliae* infection is crucial for cotton breeding [7]. In recent years, numbers of *V. dahliae* resistance genes have been detected in cotton. For example, laccase (GhLAC15) enhances *V. dahliae* resistance by increasing induced lignification and the abundance of lignin components in plant cell walls [8]. Hybrid proline-rich proteins (GbHyPRP1) negatively regulate V. wilt resistance through cell wall thickening and accumulation of reactive oxygen species (ROS) in cotton [9]. Wall-associated kinases (GhWAK7A) mediate the response to fungal pathogens by binding to chitin receptors [10]. The WAK-like kinase (GhWAKL) protein and DnaJ protein interact and participate in cotton plants against V. wilt infection [11]. Polygalacturonase inhibition protein (GhPGIP1) significantly improves cotton resistance to V. wilt [12]. U-boxE3 ubiquitin ligase (GhPUB17) interacts with the antifungal protein GhCyP3 to negatively regulate cotton’s resistance to V. wilt [13].

Rab GTPases are small GTPases [14] that play important roles in membrane recognition, vesicle movement and fusion, and intracellular vesicle transport [15]. In addition, Rab genes also respond to biotic and abiotic stress responses [16]. For example, the overexpression of the *StRab* gene can enhance resistance to potato late blight [17]. *TaRab18* positively regulates resistance to wheat stripe rust by regulating allergic responses [18]. RabG3b participates in the death of immune-related allergic cells by activating autophagy [19]. Rab5 GTPase can activate and mediate plants’ immune responses to pathogen invasion [20]. In addition, an important feature of innate immunity is phagocytosis, and Rab GTPases can coordinate and transport phagocytic substances, which enhances immunity [21]. These findings show that *Rab* genes are involved in plant immunity and hypersensitive responses.

Prenylated Rab acceptor 1 (PRA1) is a group of small transmembrane proteins. As a receptor of Rab GTPases, PRA1 primarily regulates vesicular transport [22]. The first member of PRA1 was detected in yeast as a Ypt1-interacting protein [23]. Subsequently, many proteins similar to yeast Yip/PRA1 were found in mammals and higher plants [24]. The PRA1 protein can affect the membrane localization of Rab protein by interacting with the GDP dissociation inhibitor (GDI) [25], thereby promoting the transport of small GTPases through the intimal system [26]. AtPRA1.B6 is located in the Golgi apparatus and plays a role in transporting proteins [27]. OsPRA1 is located in the prevacuolar compartment and interacts with OsRab7 to participate in the transport of plant vacuoles [28]. In addition to their functions in vesicle transport, PRA1 proteins have recently been reported to be involved in plant immunity. For example, overexpression of the *SlPRA1A* gene reduces the expression of receptor-like protein (RLP-PRR) and regulates its transport and degradation together with *AtRABA1e*, thereby weakening the immune response [29]. As PRA1 is a receptor of Rab GTPases, PRA1–Rab interactions participate in the transport of plant-related proteins and attenuate the immune response by degrading LeEIX2 pattern-recognition receptors (PRRs) in *SlPRA1A/Rab*, indicating that they may have closely related functions [29]. To date, no reports have described the role of the PRA1 protein in cotton plants, particularly in defense responses to pathogens.

To explore the potential role of PRA1, we comprehensively analyzed *GhPRA1* members in upland cotton. Additionally, *GhPRA1.B1-1A* was functionally analyzed by overexpression in *Arabidopsis thaliana* and virus-induced gene silencing (VIGS) in upland cotton. The results describe the characteristics of *GhPRA1* members in upland cotton and highlight the potential role of *GhPRA1.B1-1A* in *V. dahliae* resistance.

## 2. Materials and Methods

### 2.1. Phylogenetic Analysis

Hidden Markov model (HMM) data for PRA1 (PF03208) were downloaded from the Pfam website (http://pfam.xfam.org/, accessed on 20 September 2021), and shared homologies with upland cotton proteins were determined using the threshold value of 1E-5. The *PRA1* genes of *G. arboreum* [30], *G. raimondii* [31], *G. barbadense* [32], and *G. hirsutum* [33] (containing PRA1 domains) were detected by BLAST and regarded as *PRA1* family members. The nomenclature of PRA1 genes was according to the corresponding orthologs in *Arabidopsis thaliana* and the chromosomal location in cotton. Meanwhile, members of PRA1 in tetraploid cotton species were named according to the homologous relationship in each subgenome, for which ‘A’ and ‘D’ at the end of each gene represent the homologs in the At and Dt subgenomes, respectively.

The protein sequences of PRA1 family members in cotton and *A. thaliana* were downloaded from the Cotton Gen [34] and TAR10 (https://www.arabidopsis.org/, accessed on 22 September 2021) databases, respectively. The amino acid sequences of the PRA1 proteins were aligned using the MUSCLE program. Next, a phylogenetic tree was constructed by using MEGA-X software via the neighbor-joining method.

### 2.2. Analysis of Gene Structures, Domains, and Motifs

The Gene Structure Display Server (GSDS) was used to display the exon–intron structure of the *PRA1* genes according to their genome datasets [35]. The conserved domain of PRA1 protein was identified using the Simple Modular Architecture Research Tool [36] and was displayed using TBtools software [37]. The conserved motif of the PRA1 protein was identified using the MEME program [38].

### 2.3. Analysis of Cis-Acting Elements in the GhPRA1 Promoter Region

The sequences 2 kb upstream of the coding region of the *GhPRA1* gene were extracted using TBtools software. The sequences were uploaded to the PlantCare website to predict *cis*-acting elements, which were graphically displayed using TBtools software.

### 2.4. Collinearity Analysis of the GhPRA1 Gene

The genomic location information for *GhPRA1* was extracted from a downloaded genome annotation file, collinear gene analysis was conducted using MCScanX algorithm [39] software, and graphic visualization was conducted using Circos software [40].

### 2.5. Plant Materials and Growth Conditions

Upland cotton (*G. hirsutum*) cv. Zhongzhimian-2 seeds were soaked in distilled water for 12 h in a 28 °C incubator and then planted in soil. Cotton seedlings were grown in a greenhouse under 60% relative humidity with a dark/light cycle of 8 h in the dark (temperature: 23 °C and 16 h in the light (temperature: 28 °C).

The Colombian ecotype of *A. thaliana* was used in our experiment. *Arabidopsis* seeds were sterilized in 75% ethanol for 2 min, sterilized further in absolute ethanol for 2 min, and then sown on 1/2 MS medium for 10 days. The *Arabidopsis* seedlings were then transplanted to nutrient-supplemented soil and cultured under a 16/8 h light/dark cycle at 22/20 °C (light/dark) at 60% relative humidity.

### 2.6. Gene Cloning

Total RNA was extracted from the root tissues of Zhongzhimian 2 plants using the RNAprep Pure Plant Plus Kit (TIANGEN, Beijing, China). After quality testing, the PrimeScript RT Reagent Kit (TaKaRa, Dalian, China) was used for first-strand complementary DNA (cDNA) synthesis, according to the manufacturer’s instructions. The coding sequence (CDS) of *GhPRA1.B1-1A* was downloaded from the Cotton Gen Database, and specific primers were designed using SnapGene. The sequences of the specific primers are listed in Table S1. The full-length CDS of *GhPRA1.B1-1A* was cloned using the ClonExpress MultiS One Step Cloning Kit (Vazyme, Nanjing, China) and Zhongzhimian 2 cDNA as a template.

### 2.7. Real-Time Quantitative Polymerase Chain Reaction (RT-qPCR) Analysis

RT-qPCR analysis was performed using ChamQ Universal SYBR qPCR Master Mix (Vazyme, Nanjing, China), according to the manufacturer’s recommended protocol, on a Light Cycler 480 system machine (Roche, Basel, Switzerland). The reactions were developed in a volume of 20 μL containing 1 μL cDNA, 1 μL forward primer (10 µM), 1 μL reverse primer (10 µM), 10 µL 2× ChamQ Universal SYBR Green Master Mix, and 7 μL nuclease-free water. The reaction conditions were as follows: 40 cycles of 95 °C for 30 s, 95 °C for 10 s, and 60 °C for 15 s, followed by 95 °C for 15 s, 60 °C for 60 s, and 95 °C for 15 s. In the experiments, *GhUBQ7* was used as an internal reference gene, and three biological replicates were performed. Relative expression levels were determined using the 2^−∆∆T^ method [41].

### 2.8. Generating Transgenic Arabidopsis Plants

The full-length CDS of *GhPRA1.B1-1A* was amplified and inserted into the pCAMBIA2300 vector behind a CaMV 35S promoter to yield a recombinant vector, referred to as the p2300-*GhPRA1.B1-1A* vector. After transformation into the GV3101 *Agrobacterium*, genetic *Arabidopsis* transformants were obtained using the floral dip method [42]. Positive *Arabidopsis* seedlings were screened on 1/2 MS medium containing kanamycin and verified by PCR analysis. The T_2_ lines (in accordance with the 3:1 segregation ratio) were thought to contain single-copy insertions and were selected to observe *GhPRA1.B1-1A* expression levels by RT-qPCR. Transgenic *Arabidopsis* seedlings in the T_3_ generation were used for *V. dahliae* infection.

### 2.9. VIGS and V. dahliae Inoculation

Tobacco rattle virus was used in this study [43]. A specific 300 bp fragment of *GhPRA1.B1-1A* was inserted into the pTRV2 vector to generate the recombinant pTRV-*GhPRA1.B1-1A* vector, which was transformed into the *A. tumefaciens* strain GV3101. When both cotyledons of the cotton seedlings were fully expanded, *Agrobacterium* cultures containing pTRV1 and pTRV-*GhPRA1.B1-1A* at a 1:1 ratio (*v/v*) were transformed into cotton leaves using an injector. The chlorophyll gene *CLA1* was used as a positive control, and cotton seedlings were injected with a mixture of *Agrobacterium* containing the empty pTRV1 and pTRV2 vectors as a negative control. The VIGS assay was performed three times, and at least 48 plants were used for each experiment.

Vd991, a highly aggressive defoliating *V. dahliae* strain, was inoculated on potato dextrose agar medium and incubated at 25 °C for 7 days. The hyphae were then inoculated in potato dextrose broth medium and cultured in a shaker at 25 °C and 180 rotations/min for 5 days. The cotton seedlings were immerged in a Vd991 spore suspension (1 × 10^7^ spores mL^−1^) for 15 min. The method of *A. thaliana* infection by *V. dahliae* was according to the method of Zhao et al. [44]. The disease index and incidence rate were calculated 2 weeks after inoculation, based on the disease index (DI) classification [45].

### 2.10. Plant Disease Evaluation

According to the severity of the diseased phenotype in true cotton leaves, the DI was graded from 0 to 4, using the following formula: DI = (∑[incidence grade × number of plants at this level]/[total number of plants investigated × 4]) × 100 [46]. The lower the DI value, the lower the degree of disease in the plant. In addition, fungal restoration experiments and fungal biomass testing were performed to determine the degree of plant disease [47].

### 2.11. ROS and Callose Deposition Detection

After inoculation for 24 h, the true leaves were washed with distilled water, placed in a 50 mL centrifuge tube, and stained with a 3,3′-diaminobenzidine staining solution. After incubation at 25 °C for 8 h and destaining with 95% ethanol, the leaves were observed under a microscope and photographed. At least five seedlings were detected in each treatment.

For callose deposition, cotton leaves were sampled 2 weeks after inoculation. The leaves were decolorized in a 3:1 mixture of ethanol and acetic acid for 3 h, soaked sequentially in 70% and 50% ethanol for 2 h, and then soaked in water overnight. After treatment with 10% NaOH for 2 h, callose was stained with 0.01% aniline blue dye for 4 h. Next, the callose content was observed by ultraviolet excitation light under a fluorescence microscope.

### 2.12. Measuring Jasmonic Acid, Salicylic Acid, and H_2_O_2_ Content

The leaves of TRV:00 and TRV:*GhPRA1.B1-1A* plants were obtained at 48 h after inoculation, and the contents of JA and SA were measured by high-performance liquid chromatography. Three biological replicates were used for each experiment.

H_2_O_2_ was measured in the leaves of TRV:00 and TRV:*GhPRA1.B1-1A* plants inoculated with *V. dahliae* for 48 h using the H_2_O_2_ Kit (Solarbio; Beijing, China), according to the manufacturer’s instructions.

### 2.13. Statistical Analysis

GraphPad Prism8 software was used for constructing graphs, and the t-test was used to analyze statistical differences. “*” and “**” indicate significances at *p*-values of <0.05 and <0.01, respectively.

## 3. Results

### 3.1. Identification and Phylogenetic Analysis of PRA1 Family Members

**Figure 1:**
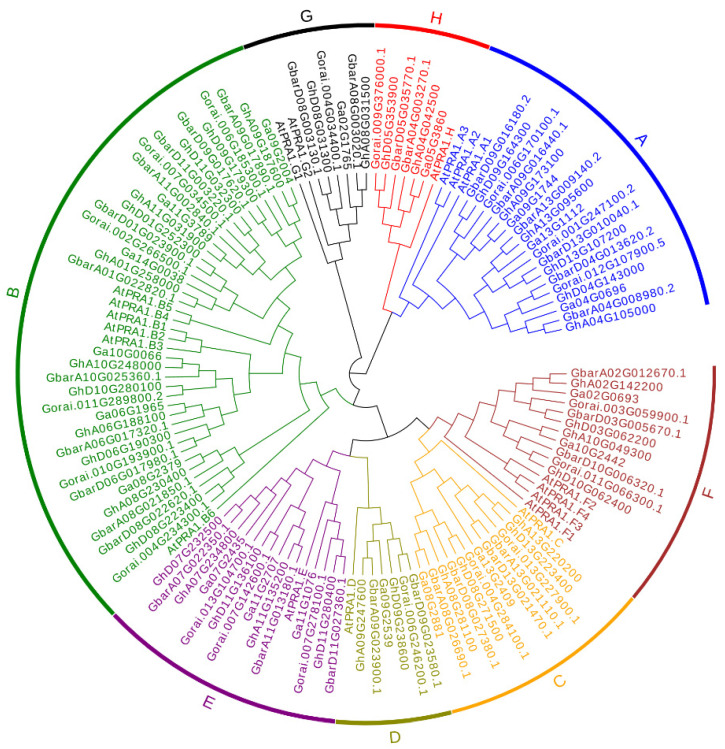
Phylogenetic analysis of the PRA1 gene family. At, *A. thaliana*; Ga, *G. arboreum*; Gr, *G. raimondii*; Gb, *G. barbadense*; Gh, *G. hirsutum*.

To understand the evolutionary relationship of the PRA1 gene family in *G. hirsutum*, *G. raimondii*, *G. arboreum,* and *G. barbadense*, the conserved domains of PRA1 in the four cotton species were detected by Pfam (PF03208). An evolutionary tree was constructed according to the amino acid sequence of PRA1 protein. (Figure 1). The PRA1 family can be divided into eight groups. Among these, the B subfamily of PRA1 contains the most members (41), has the farthest evolutionary relationship with the F subfamily, and has the closest evolutionary relationship with the E and G subfamilies. We observed that the number of PRA1 genes in tetraploid cotton species (*G. hirsutum* and *G. barbadense*) was approximately twice as much as that in diploid cotton (*G. arboreum* and *G. raimondii*). Meanwhile, PRA1 genes in the At subgenomes and A_2_ genome (*G. arboreum*) tended to form one clade, and PRA1 genes in the Dt subgenomes and D_5_ genome (*G. raimondii*) tended to form another clade. These results confirm that the tetraploid cotton species were a result of the hybridization of diploid cottons, followed by a genome doubling event during cotton’s evolution. Additionally, the phylogenetic tree showed that PRA1 members in each group were conserved in the four cotton species and *A. thaliana*, indicating that PRA1 family members exhibit conserved sequences in dicotyledons.

Thirty-seven *PRA1* members were identified in upland cotton, and detailed information is presented in Table 1. The CDSs of the 37 *PRA1* genes were between 291 bp (*GhPRA1.E-1D*) and 801 bp (*GhPRA1.G2-1A*) in length. The molecular weight (MW) of PRA1 proteins ranged from 11.18 kDa (GhPRA1.E-1D) to 28.99 kDa (GhPRA1.G2-1A), and the isoelectric point ranged from 4.59 (GhPRA1.D-2D) to 10.84 (GhPRA1.E-1D). Subcellular localization analysis revealed that 14 of the PRA1 proteins were predicted to be located in the vacuoles, 12 PRA1 proteins were predicted to be located in the plasma membrane, nine PRA1 proteins were predicted to be located in the chloroplasts, and two PRA1 proteins were predicted to be located in the endoplasmic reticulum. The full-length 657 bp sequence of the *GhPRA1.B1-1A* gene encodes 218 amino acids, with an MW of 23.313 kDa and a theoretical isoelectric point of 9.566.

### 3.2. Analysis of the Gene Organization and Conserved Domains of GhPRA1s

**Figure 2:**
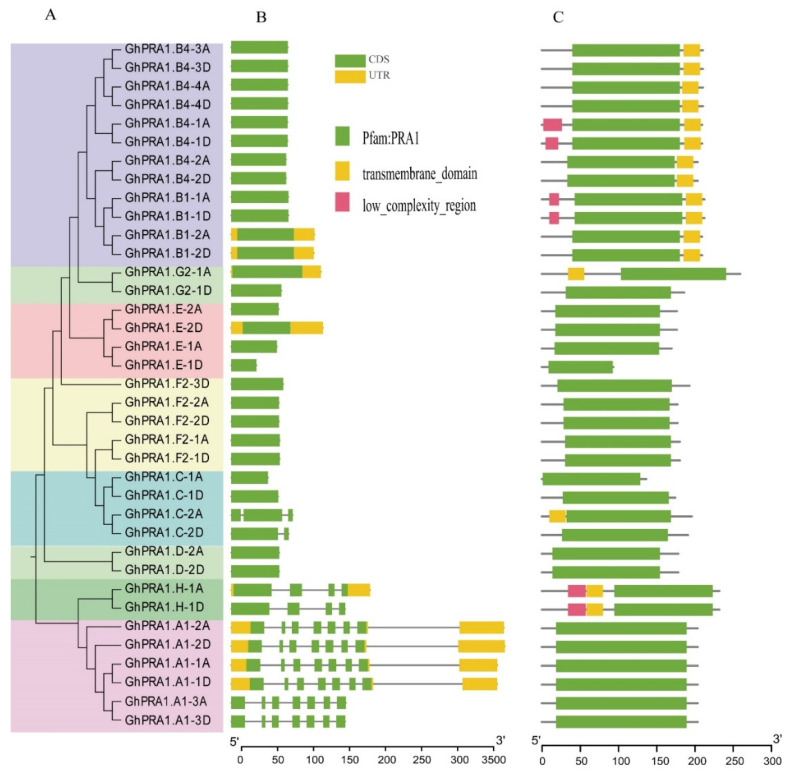
Gene structures and conserved domains in GhPRA1 family members. (**A**) Phylogenetic tree of GhPRA1 proteins. (**B**) The organization of exons and introns in *GhPRA1* genes. (**C**) Conserved domains in GhPRA1 proteins.

To understand the phylogenetic relationship of the PRA1 members in upland cotton, a phylogenetic tree was constructed using the 37 *GhPRA1* amino acid sequences (Figure 2A). The results showed that the PRA1 proteins also can be divided into eight groups. PRA1 proteins in the same group were clustered together and shared high sequence identity. The exon–intron structure of PRA1 genes was analyzed using the online tool GSDS (http://gsds.gao-lab.org/, accessed on 27 September 2021). We observed that members of Subfamily A had the longest gene length and contained seven exons (Figure 2B). However, most *GhPRA1* members in the other subfamilies were found to have only one exon, with a highly similar gene structure in the same subfamily.

In addition, the conserved domain of GhPRA1 was identified. All GhPRA1 members contained a PRA1 domain, and we also observed that GhPRA1.B1 group members, GhPRA1.B group members, GhPRA1.G2-1A, GhPRA1.C-2A, and GhPRA1.H-1A/D contain a transmembrane domain (Figure 2C). Furthermore, the PRA1 protein motif was analyzed using MEME software, and the top 10 most conserved motifs were presented. The results showed that all GhPRA1 proteins contain Motif 1. We also found that most homologous proteins have similar motifs and show highly conserved structures (Figure S1). Both GhPRA1.B1 and GhPRA1.B4 have Motifs 2 and 9. Except for GhPRA1.H and GhPRA1.A1, all the other GhPRA1 proteins were found to have Motifs 3 and 4. Only GhPRA1.A1 contains Motifs 5, 6, and 7. All GhPRA1 proteins contain Motif 8, except for GhPRA1.B1, GhPRA1.B4 and GhPRA1.A1. The motif organization of the GhPRA1 members was based on a phylogenetic tree, which indicated evolutionary relationships. The function of motifs was detected by the Pfam website, showing that Motif 1, Motif 3, Motif 4, Motif 7, and Motif 8 belong to the PRA1 domain, and Motif 6 was part of the Popeye protein’s conserved region (Table S2).

### 3.3. Analysis of Cis-Acting Elements of PRA1 Promoter

**Figure 3:**
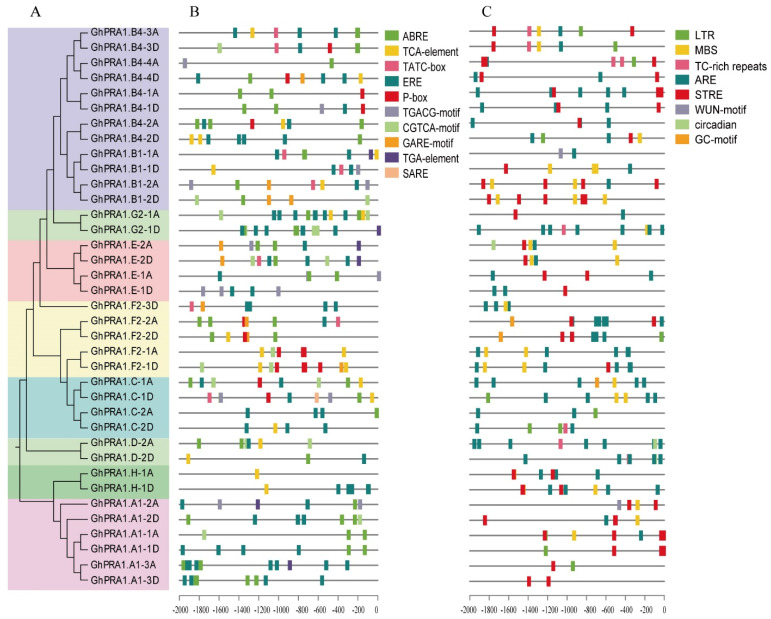
*cis*-elements in GhPRA1 promoter regions. (**A**) Phylogenetic tree of GhPRA1 proteins. (**B**) Hormone response elements. ABRE, abscisic acid response element; P-box; TATC box; GARE motif, gibberellin response element; TCA element; SARE, salicylic acid responsiveness element; CGTCA motif; TGACG motif. MeJA response *cis*-regulating elements: ERE, ethylene response element; TGA element, auxin response element. (**C**) Environmental stress related elements: STRE, stress response element; ARE, anaerobic induction element; MBS, MYB drought induction binding site; LTR, low temperature response element; GC motif, anoxic specific inducibility enhancer-like element; circadian, *cis*-acting regulatory element involved in circadian rhythm regulation; WUN motif, wound-responsive element; TC-rich repeats, defense and stress response *cis*-elements.

The phylogenetic tree showed that the 37 GhPRA1 members were divided to eight groups (Figure 3A). The 2 kb promoter regions upstream of the initiation codons of *GhPRA1* genes were used to scan for *cis*-acting elements. *Cis*-acting elements (Table S3) in the *GhPRA1* promoter regions were predicted to facilitate different functions, including anaerobic induction, meristem expression, hormone production, and stress defense. In particular, numbers of *cis*-elements related to hormones were predicted in the *GhPRA1* promoter regions, such as ethylene (ET), abscisic acid (ABA), gibberellin (GA), salicylic acid (SA), methyl jasmonate (MeJA), and auxin (IAA), among others (Figure 3B). For instance, the *cis*-acting elements ERE, ABRE, TCA element, and CGTCA motif were predicted in the promoter region of *GhPRA1.B1-1A*, indicating that *GhPRA1.B1-1A* may be involved in ET, JA, and SA signal transduction pathways. In addition, *cis*-elements participated in environmental adaptation, such as ARE, STRE, MBS, LTR, the WUN-motif, the GC motif, and TC-rich repeats, and were also predicted in the *GhPRA1* promoter regions (Figure 3C). The result suggest that the *GhPRA1* family may play a role in drought, low temperature, defense, and stress responses.

### 3.4. Chromosomal Location and Gene Synteny Analyses of GhPRA1 Genes

**Figure 4:**
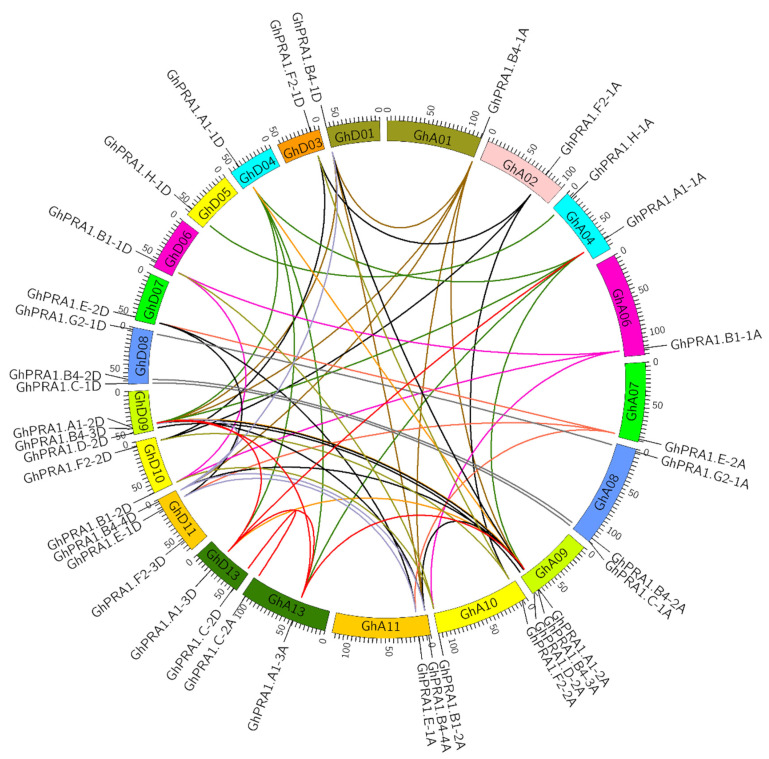
Syntenic relationships of *GhPRA1* genes in upland cotton. Homologous chromosomes in the At and Dt subgenomes are displayed in the same color. The brown lines represent repetitive genes of *GhPRA1.B4-1A/D*; dark blue lines represent repetitive genes of *GhPRA1.F2-1A/D*; the green lines indicate repetitive genes of *GhPRA1.A1-1A/D* and homologous genes of *GhPRA1.H-1A*; the purple line indicates a repetitive gene of *GhPRA1.A1-1A/D*; the pink line indicates a repetitive gene of *GhPRA1.E-2A*; the gray line indicates a homologous gene of *GhPRA1.G2-1A* Magi *GhPRA1.C-1A;* the orange line represents a repetitive gene of *GhPRA1.A1-2A/D;* the black line represents a repetitive gene of *GhPRA1.B4-3A/D;* and the light brown line represents a repetitive gene of *GhPRA1.C-1A*. With respect to the repetitive genes in *GhPRA1.B1-2A* and *GhPRA1.F2-2A*, the light purple line indicates a repetitive gene of *GhPRA1.B4-4A 3A/D*, and the red line indicates a repetitive gene of *GhPRA1.A1-3A/D* and the homologous gene in *GhPRA1.E-3A*.

We conducted chromosomal location and gene synteny analyses of the *GhPRA1* genes. The results showed that most of the *GhPRA1* members were highly symmetrical between the At and Dt subgenomes. *GhPRA1* genes were unevenly distributed on all chromosomes, except for chromosomes A03, A12, D02, and D12. However, *GhPRA1.F2-1A* and *GhPRA1.H-1A* were located on chromosomes A02 and A04, respectively, but the homologous genes *GhPRA1.F2-1D* and *GhPRA1.H-1D* were located on chromosomes D03 and D05, respectively (Figure 4).

Duplicated GhPRA1 genes were analyzed in this study. We observed that GhPRA1.B4-1A/1D, GhPRA1.B4-3A/3D, and GhPRA1.B4-4A/4D; GhPRA1.F2-1A/1D and GhPRA1.F2-2A/2D; GhPRA1.A1-1A/1D, GhPRA1.A1-3A/3D, and GhPRA1.A1-2A/2D; GhPRA1.B1-1A/1D and GhPRA1.B1-2A/2D; and GhPRA1.E-2A/2D and GhPRA1.E-1A/1D may be duplicated genes. Based on the locations of the duplicated genes, these genes might have resulted from segmental duplication. The large number of duplicated genes suggested that the GhPRA1 genes duplicated frequently during upland cotton evolution.

### 3.5. Expression Profiles of GhPRA1 in Upland Cotton

**Figure 5:**
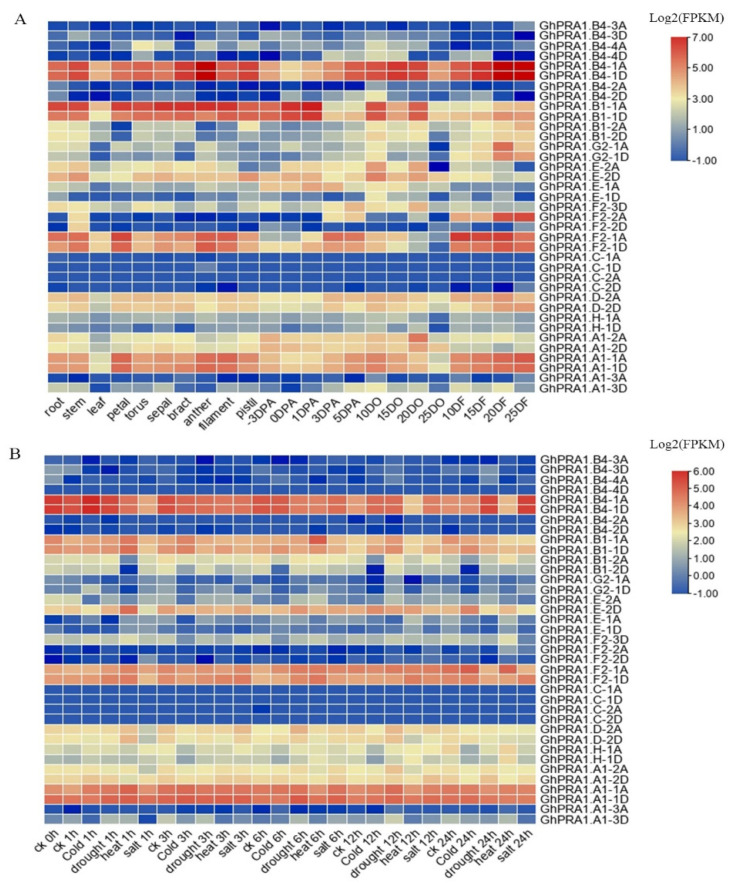
Expression patterns of *GhPRA1* genes in different tissues under abiotic stress in upland cotton. (**A**) The expression profiles of *GhPRA1* genes in cotton tissue. DO and DF represent DPA ovules and fibers, respectively. (**B**) The expression profiles of *GhPRA1* genes under abiotic stress.

To have insights into the functions of *GhPRA1* genes, transcriptome datasets [48] were used to analyze *GhPRA1* expression patterns in different tissues during the development of upland cotton. We observed that *GhPRA1.B4-3A/D*, *GhPRA1.B4-2A*, *GhPRA1.C-1A/D*, *GhPRA1.C-2A/D,* and *GhPRA1.A1-3A* were barely expressed in upland cotton tissues. However, *GhPRA1.B1-1A/D* was highly expressed in roots, stems, petals, the torus, sepals, bracts, anthers, filaments, and early developmental ovules, indicating that *GhPRA1.B1-1A/D* might be involved in the growth and reproductive development of cotton. The finding that *GhPRA1.B4-1A* was abundantly expressed in anthers and fibers further suggested that *GhPRA1.B4-1A* may be related to reproductive development and fiber elongation. We also found that *GhPRA1.F2-2A* and *GhPRA1.F2-1A/D* were predominantly expressed in 25 DPA fibers, suggesting their roles in fiber growth (Figure 5A).

We also investigated the expression profiles of *GhPRA1* genes under abiotic stresses, including cold, heat, drought, and salt stress (Figure 5B). *GhPRA1.B4-1A* expression was elevated after 1 h and 12 h of cold stress, but was decreased after 3 h of cold stress. The *GhPRA1.B4-1A* gene was downregulated after 3 h and 6 h of drought stress, and was upregulated after 12 h and 24 h of exposure to drought conditions. *GhPRA1.B4-1A* exhibited decreased expression after 1 h, 3 h, 6 h, 12 h, and 24 h of heat stress. The *GhPRA1.B4-1A* gene was downregulated after 1 h, 3 h, and 6 h of salt stress, but was upregulated after 12 h and 24 h of exposure to saline conditions. The expression of *GhPRA1.B1-1A* was elevated after 12 h of cold and drought stress, but decreased after 24 h. The *GhPRA1.B1-1A* genes were abundantly expressed after 1 h and 6 h of exposure to heat conditions, but decreased after 24 h. The *GhPRA1.B1-1A* gene was downregulated after 3 h and 24 h of salt stress. The expression of *GhPRA1.B1-1D* was increased after 3 h, 6 h, 12 h, and 24 h of cold stress. The *GhPRA1.B1-1D* genes were abundantly expressed after 1 h and 6 h of exposure to heat conditions. The *GhPRA1.E-2D* gene was downregulated after 1 h and 3 h of cold stress, and upregulated after 6 h, 12 h, and 24 h of exposure to cold conditions. The *GhPRA1.F2-1A* genes were abundantly expressed after 6 h and 24 h of exposure to cold conditions. The *GhPRA1.F2-1A* gene showed increased expression after 1 h, 6 h, and 24 h of heat stress. The *GhPRA1.F2-1D* genes were abundantly expressed after 3 h, 6 h, and 12 h of exposure to salt conditions. These results suggest that *GhPRA1* genes may participate widely in cotton’s adaptation to abiotic stresses.

**Figure 6:**
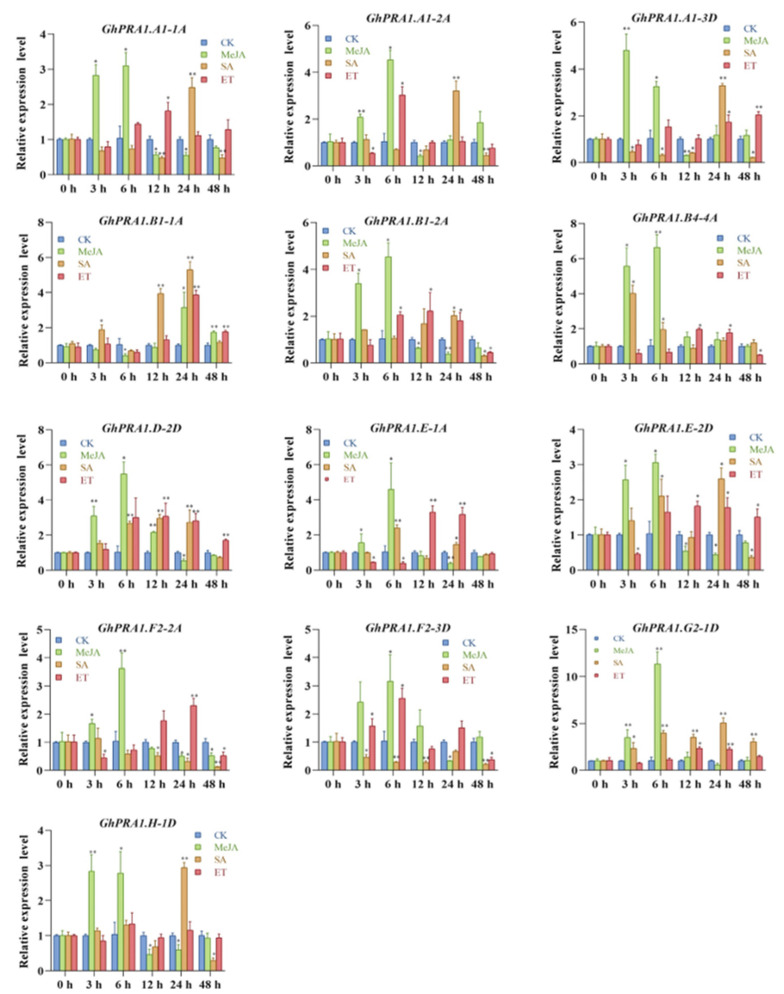
Expression profiles of *GhPRA1* genes under treatment with different plant hormones. Statistically significant differences are indicated as follows: * *p* < 0.05, ** *p* < 0.01.

Plant hormone-mediated signal transduction is an important process in plant immune responses, and JA, SA, and ET have been found to play an important role in biological stress signals. Thus, we examined the effects of MeJA, MeSA, and ET treatments on *GhPRA1* expression by the RT-qPCR approach (Figure 6). We observed that the transcriptional levels of the *GhPRA1.B1-1A* gene were upregulated at 24 h and 48 h after MeJA treatment. Most *GhPRA1* genes were downregulated at 48 h after exposure to MeJA and SA. The expression levels of *GhPRA1.F2-2A* and *GhPRA1.F2-3D* decreased at multiple time points after SA and at 48 h after ET treatment, whereas those of *GhPRA1.B1-1A* and *GhPRA1.D-2D* increased. *GhPRA1.D-2D*, *GhPRA1.E-1A GhPRA1.E-2D,* and *GhPRA1.G2-1D* were induced at 12 h and 24 h after ET, and at 6 h and 24 h after SA treatment. These results show that the *GhPRA1* genes responded to MeJA, SA, and ET treatments, and that *GhPRA1* genes may be involved in plant immunity.

### 3.6. Overexpression of GhPRA1.B1-1A Enhanced V. dahliae Resistance in Arabidopsis

**Figure 7:**
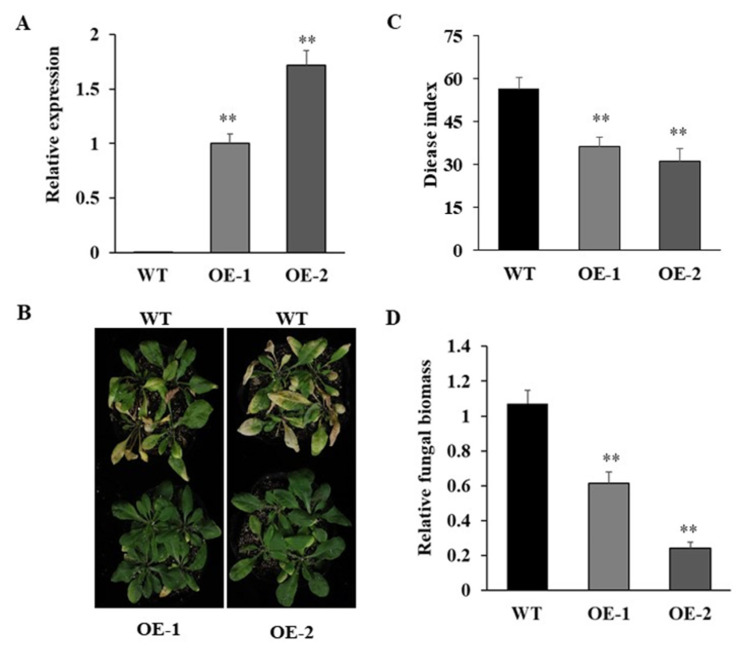
*GhPRA1.B1-1A* overexpression increased *V. dahliae* resistance in *A. thaliana*. (**A**) The transcriptional levels of *GhPRA1.B1-1A* in OE and wild-type lines were detected by RT-qPCR. (**B**) The *V. dahliae*-resistant phenotype of wild-type and transgenic plants after inoculation with Vd991. (**C**) Statistical analysis of the plants’ DI. (**D**) Fungal biomass in rosette leaves of *A. thaliana*, as detected by RT-qPCR. T-tests were performed to identify statistically significant differences (** *p* < 0.01). All experiments were repeated at least three times.

The expression patterns of *GhPRA1* genes responding to *V. dahliae* infection were investigated by using public RNA-Seq data, in which only the expression of the homologous genes *GhPRA1.B1-1A* and *GhPRA1.B1-1D* were significantly induced (Figure S2). Meanwhile, the upregulated expression of *GhPRA1.B1-1A* upon Vd991 infection was confirmed by RT-qPCR (Figure S3). These results suggest that *GhPRA1.B1-1A* may play a defensive role in cotton against Vd991 infection. To further investigate the role of *GhPRA1.B1-1A* in *V. dahliae* resistance, the p2300-*GhPRA1.B1-1A* overexpression vector driven by the 35S promoter was transformed into *A. thaliana* by the flower dipping method. A single-copy insertion transgenic line with high *GhPRA1.B1-1A* expression in the T_3_ generation was used for *V. dahliae* infection. The results showed that *V. dahliae* resistance was enhanced in *GhPAR1.B1-1A* overexpressing (OE) lines (Figure 7B,C). In addition, the fungal biomass of the transgenic lines was significantly lower than that of the wild-type (Figure 7D). Therefore, these results indicate that *GhPRA1.B1-1A* overexpression improved *V. dahliae* resistance in *A. thaliana*.

### 3.7. Silencing GhPRA1.B1-1A Reduced V. dahliae Resistance in Upland Cotton

**Figure 8:**
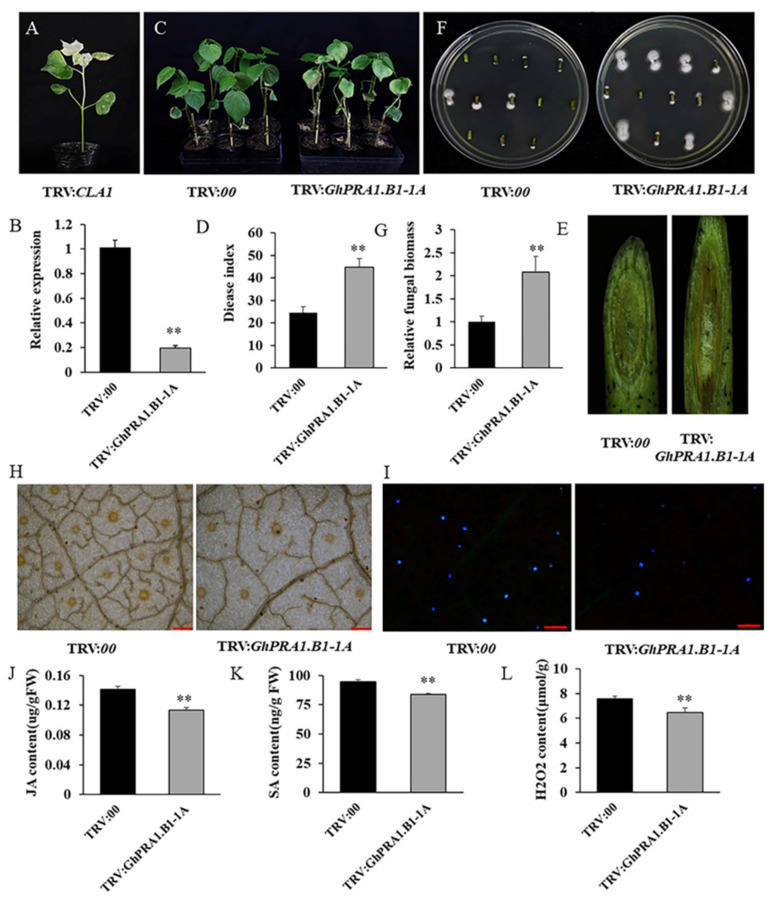
Silencing *GhPRA1.B1-1A* weakened *V. dahliae* resistance in upland cotton plants. (**A**) The photobleached phenotype after infection by TRV:*GhCLA1*. (**B**) Detection of the expression levels of the *GhPRA1.B1-1A* gene in *GhPRA1.B1-1A*-silenced plants. (**C**) Phenotypes of control (TRV:00) and TRV:*GhPRA1.B1-1A* plants. (**D**) DI after 21 days of Vd991 infection. (**E**) Observation of the browning degree in stem vascular bundles. (**F**,**G**) The degree of fungal colonization and relative fungal biomasses of TRV:00 and TRV:*GhPRA1.B1-1A*. (**H**) At 48 h after inoculation, the levels of ROS were detected by DAB; bar = 200 μm. (I) Callose deposition in TRV:00 and TRV:*GhPRA1.B1-1A* cotton leaves; bar = 200 μm. (**J**) JA contents of TRV:00 and *TRV:GhPRA1.B1-1A*. (**K**) SA contents of TRV:00 and *TRV:GhPRA1.B1-1A*. (**L**) H_2_O_2_ contents of TRV:00 and *TRV:GhPRA1.B1-1A*. The difference was statistically significant (** *p* < 0.01).

To determine the function of *GhPRA1.B1-1A* in *V. dahliae* resistance in upland cotton, the VIGS approach was used to downregulate *GhPRA1.B1-1A* expression. The pTRV2 vector (TRV:*GhPRA1.B1-1A*) was constructed with a specific fragment of *GhPRA1.B1-1A*, and the empty pTRV2 vector was used as a negative control (TRV:00). When photobleaching was observed in the positive control (TRV:*CLA*) (Figure 8A), RT-qPCR was used to detect *GhPRA1.B* expression in the silenced plants. The observation of *GhPRA1.B1-1A* downregulation suggested that *GhPRA1.B1-1A* was effectively silenced in cotton plants (Figure 8B). However, the expression of other members in *GhPRA1.B* subgroup were not affected by *GhPRA1.B1-1A* silencing, except for *GhPRA1.B1-1D* (the homologous gene of *GhPRA1.B1-1A*) (Figure S4). The plants were then infected with Vd991, and *V. dahliae* resistance was weakened in *GhPRA1.B1-1A*-silenced cotton plants, in which the leaves showed more serious yellowing and wilting phenotypes than the negative control (Figure 8C,D). In addition, the vascular bundles in *GhPRA1.B1-1A*-silenced plants were more browned than those in the negative control (Figure 8E). Fungal recovery experiments and fungal biomass detection showed that the fungal contents of TRV:*GhPRA1.B1-1A* samples were higher than those of TRV:00 (Figure 8F,G). Enhanced ROS production and callose deposition have been confirmed as important defense responses of plants to pathogen invasion [49]. Therefore, cotton leaves in the TRV:00 and TRV:*GhPRA1.B1-1A* plants were stained with DAB, and ROS levels were determined. We found that ROS accumulation and H_2_O*_2_* levels in *GhPRA1.B1-1A*-silenced plants were significantly lower than in control plants (Figure 8H,L). In addition, less callose deposition was observed in *GhPRA1.B1-1A*-silenced cotton plants than in control plants (Figure 8I). These results indicated that *V. dahliae* resistance was weakened by downregulating the expression of *GhPRA1.B1-1A* in upland cotton.

### 3.8. GhPRA1.B1-1A Affected PR Genes’ Expression and Regulated the SA and JA Signaling Pathways

**Figure 9:**
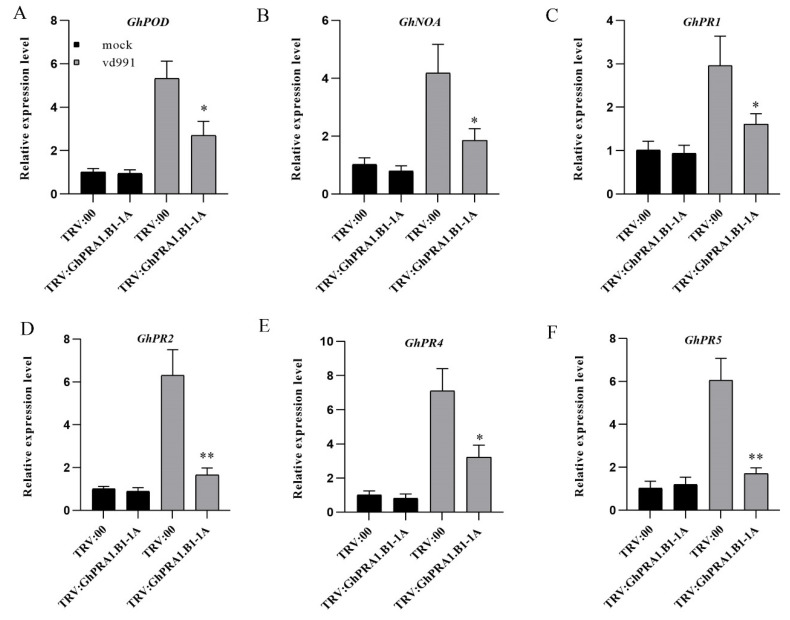
Expression levels of *PR* genes in *GhPRA1.B1-1A*-silenced cotton. (**A**–**F**), RT-PCR analysis of the expression levels of PR genes in silenced plants and controls. All experiments were repeated at least three times (* *p* < 0.05, ** *p* < 0.01).

To study the molecular basis whereby *GhPRA1.B1-1A* participates in *V. dahliae* resistance, the expression patterns of several *PR* genes were detected in *GhPRA1.B1-1A*-silenced cotton plants. In particular, the expression levels of *GhPR2* and *GhPR5* decreased threefold in the silenced plants. The expression levels of many *PR* genes in TRV:*GhPRA1.B1-1A* plants were significantly downregulated compared with the control plants, suggesting that *GhPRA1.B1-1A* affects the expression of PR genes (Figure 9).

*GhPRA1.B1-1A* was induced by SA and MeJA treatments (Figure 6); thus, we determined the contents of SA and JA in TRV:00 and TRV:*GhPRA1.B1-1A* cotton leaves 48 h after inoculation with *V. dahliae*. We observed that the JA and SA contents in TRV:*GhPRA1.B1-1A* cotton seedlings were significantly lower than those in TRV:00 plants (Figure 8J,K). Therefore, our results indicated that *GhPRA1.B1-1A* promotes *V. dahliae* resistance via the SA and JA pathways.

## 4. Discussion

Pattern recognition receptors can trigger plant immune responses by recognizing microbe-related molecular patterns [50]. The recognition and transmission of pathogens by PRRs depends on transport to the plasma membrane [51]. Rab proteins play an important role in plant immunity [18,19,20,21], and the PRA1 protein regulates Rab proteins and mediates their transport by stabilizing their localization on the cell membrane [25]. The findings of this study expounded upon the role of *GhPRA1* in cotton’s resistance to *V. dahliae* and provided a basis for functional analysis of the cotton *PRA1* gene.

### 4.1. Evolution of GhPRA1 Genes

The *PRA1* gene family has been identified in *A. thaliana* [22]. However, *PRA1* gene members have not been identified or analyzed in cotton. In the present study, we analyzed the evolutionary relationship and expression profiles of *PRA1* genes in upland cotton. The allotetraploid species of *G. hirsutum* and *G. barbadense* resulted from the hybridization of diploid cotton *(G. arboreum* and *G. raimondii*) [52]. We identified 108 *PRA1* genes in *G. arboreum, G. raimondii*, *G. hirsutum*, and *G. barbadense*. Approximately twice as many *PRA1* genes were identified in the tetraploid cotton species *G. hirsutum* (37 genes) and *G. barbadense* (33 genes) than in the diploid cotton species *G. arboreum* (19 genes) and *G. raimondii* (19 genes)*,* which provides further evidence that the allotetraploid cotton species were derived from the hybridization of two diploid species.

PRA1 proteins can be divided into eight subfamilies according to phylogenetic analysis. The GhPRA1 proteins in the same subfamily clustered together and showed a high sequence identity. Furthermore, the *GhPRA1* members in the same subfamily were found to have similar exon and intron structures. Furthermore, we found that most *GhPRA1* members have only one exon.

In addition, PRA1 proteins in the same group have conserved protein motifs, which is in accordance with the results of our gene structure analysis. The results of synteny analysis suggested that a strong synteny relationship occurred with the *PRA1* gene in upland cotton. Gene duplication analysis showed that *GhPRA1.B4-3A/3D* and *GhPRA1.B4-4A/4D; GhPRA1.A1-1A/1D* and *GhPRA1.A1-3A/3D; GhPRA1.B1-1A/1D* and *GhPRA1.B1-2A/2D;* and *GhPRA1.E-2A/2D* and *GhPRA1.E-1A/1D* have arisen through fragment duplications. Thus, fragment duplications may have promoted the evolution and expansion of *GhPRA1* family members.

### 4.2. Expression of GhPRA1 in Tissue and under Abiotic Stress

PRA1 protein is a known receptor of Rab GTPases, which mediate vesicular transport; however, the role of PRA1 in abiotic stresses has not been extensively studied. Mutant *atpr1.f4* and overexpressing *AtPRA1.F4* plants were more sensitive to salt stress [53]. Analysis of the *cis*-acting elements showed that several STRE elements were found in the *GhPRA1.B4-1A/D* and *GhPRA1.E-2D* promoter regions. Wound-responsive elements were found in *GhPRA1.A1-2A* and *GhPRA1.B1-1A*. Additionally, the expression of *GhPRA1.B4-1A* increased under drought and salt stress after 24 h. The *GhPRA1.E-2D* gene was upregulated after exposure to heat and drought treatment at 1 h and 6 h. The expression of *GhPRA1.F2-1A/D* increased after 6 h under drought and heat stress conditions. Accordingly, MYB-binding site elements were predicted in their promoters. In summary, *GhPRA1* family members may participate in environmental stress resistance in cotton plants.

### 4.3. GhPRA1.B1-1A Enhanced Cotton’s Resistance to V. dahliae through ROS Accumulation and the SA and JA Signaling Pathways

Plant hormones play vital roles in regulating plant defense responses. JA, SA, and ET are essential for transducing signals responsible for inhibiting pathogen infection [54,55]. SA is primarily involved in immunity against biological nutritional pathogens. JA plays roles in plants’ defenses against necrotizing pathogens and in the defense response to physical wounding [56]. *GbMPK3* overexpression leads to cotton being more sensitive to *V. dahliae* by regulating SA-dependent signal transduction [57]. Silencing *GbEDS1* significantly reduced SA and H_2_O_2_ accumulation and decreased the resistance of cotton to *V. dahliae* [46]. *Gbvdr6* conferred *V. dahliae* resistance by regulating the JA and SA signaling pathways [58]. The ET response-related factor (*GbERF1-like*) participates in *V. dahliae* resistance by regulating lignin synthesis [59]. *GaRPL18* participates in the SA signal transduction pathway and increases cotton’s resistance to *V. dahliae* [60]. In this study, many hormone-related elements were found in the *GhPRA1* promoters (Figure 3B), such as the ethylene-response element (ERE), the TCA element, the salicylic acid response element (SARE), the CGTCA motif, and the TGACG motif (associated with the JA response element). In particular, we detected two EREs, two JA response elements, and one SA in the *GhPRA1.B1-1A* promoter region. These results imply that *GhPRA1.B1-1A* may be involved in cotton’s responses to pathogens by regulating SA and JA signals. To test this possibility, the VIGS approach was used to silence *GhPRA1.B1-1A* in upland cotton, which revealed that TRV:*GhPRA1.B1-1A* plants were more sensitive to *V. dahliae* infection. Furthermore, the JA and SA contents in TRV:*GhPRA1.B1-1A* seedlings were significantly lower than those in TRV:00 plants. Therefore, *GhPRA1.B1-1A* may be involved in SA and JA signaling pathways to enhance *V. dahliae* resistance in cotton.

ROS are cellular signaling molecules that can recognize pathogens and trigger immune and cell death responses in plants [3]. H_2_O_2_ production by cell wall peroxidases plays a vital role in the immune responses triggered by pathogen-associated molecular patterns. Previous findings showed that indicators of ROS accumulation were upregulated in cotton roots infected with *V. dahliae*, indicating the key role of ROS in defense responses [49,61,62]. *HyPRP1* negatively regulates resistance in cotton through ROS accumulation and cell wall thickening [9], whereas *GhGPA* positively regulates *V. dahliae* resistance through H_2_O_2_ accumulation [7]. In this study, ROS accumulation and H_2_O_2_ levels were significantly lower in *GhPRA1.B1-1A*-silenced cotton than in the control (Figure 8L). These results suggest that *GhPRA1.B1-1A* protects cotton against *V. dahliae* infection through ROS accumulation. In this study, ROS and callose accumulation decreased, fungal biomasses increased, and vascular bundle browning was more serious in *GhPRA1.B1-1A*-silenced plants after *V. dahliae* inoculation, compared with the corresponding findings in the control plants. This result agrees with the toxin theory, and suggests that *GhPRA1.B1-1A* participates in *V. dahliae* resistance through the ROS pathway.

## 5. Conclusions

In the present study, we characterized *PRA1* family genes in cotton, and the particular role of *GhPRA1.B1-1A* in *V. dahliae* resistance was highlighted. *V. dahliae* resistance was increased significantly by the overexpression of *GhPRA1.B1-1A* in *A. thaliana*, whereas *V. dahliae* resistance was decreased by silencing *GhPRA1.B1-1A* in cotton. Additionally, *GhPRA1.B1-1A* might participate in *V. dahliae* resistance through the SA and JA signal transduction pathways and ROS accumulation. The findings of this study help in understanding the potential role of *GhPRA1.B1-1A* in *V. dahliae* resistance and provide a theoretical basis for breeding cotton plants resistant to V. wilt.

## Figures and Tables

**Table 1:** Identification of the *GhPRA1* genes. Abbreviations: vacu, vacuole; plas, plasmalemma; golg, Golgi; chlo, chloroplast; ER, endoplasmic; extr, extracellular. The top three most possible subcellular localizations are shown, and the location numbers indicate the possibility of subcellular localizations.

Gene Name	Gene Locus ID	Chr Location	CDS	Size	MW (kDa)	pI	Subcellular Localization
*GhPRA1.A1-1A*	Gh_A04G105000.1	A04: 70948155–70951205	630	209	24.19	10.58	vacu: 5, plas: 4, extr: 2
*GhPRA1.A1-1D*	Gh_D04G143000.1	D04: 44330508–44333557	630	209	24.20	10.44	vacu: 7, plas: 4, golg: 2,
*GhPRA1.A1-2A*	Gh_A09G173100.1	A09: 74156483–74159609	630	209	24.17	10.58	vacu: 12, plas: 1, golg: 1
*GhPRA1.A1-2D*	Gh_D09G164300.1	D09: 43855015–43858151	630	209	24.23	10.48	vacu: 11, plas: 2, golg: 1
*GhPRA1.A1-3A*	Gh_A13G095600.1	A13: 40978102–40979417	630	209	23.98	10.41	vacu: 6, plas: 4, golg: 2
*GhPRA1.A1-3D*	Gh_D13G107200.1	D13: 24914233–24915541	630	209	24.11	10.45	plas: 5, vacu: 5, golg: 2
*GhPRA1.B1-1A*	Gh_A06G188100.1	A06: 117938223–117938879	657	218	23.31	9.44	chlo: 5, ER: 4, plas: 3
*GhPRA1.B1-1D*	Gh_D06G190300.1	D06: 58492262–58492918	657	218	23.40	9.64	chlo: 7, ER: 3, plas: 2
*GhPRA1.B1-2A*	Gh_A10G248000.1	A10: 114198294–114199248	648	215	23.49	9.64	chlo: 6, ER: 3, plas: 2
*GhPRA1.B1-2D*	Gh_D10G280100.1	D10: 65324234–65325180	648	215	23.54	9.83	chlo: 6, ER: 3, plas: 2
*GhPRA1.B4-1A*	Gh_A01G258000.1	A01: 115880792–115881439	648	215	23.32	7.82	E.R.: 5, plas: 3, chlo: 2
*GhPRA1.B4-1D*	Gh_D01G252300.1	D01: 64514473–64515120	648	215	23.33	7.82	E.R.: 4, plas: 3, chlo: 2,
*GhPRA1.B4-2A*	Gh_A08G230400.1	A08: 119818691–119819320	630	209	22.74	8.68	vacu: 7, ER: 3, plas: 2
*GhPRA1.B4-2D*	Gh_D08G223400.1	D08: 63336820–63337449	630	209	22.81	8.68	vacu: 7, ER: 3, plas: 2
*GhPRA1.B4-3A*	Gh_A09G187600.1	A09: 75447349–75447999	651	216	23.70	9.03	chlo: 5, plas: 5, golg: 2
*GhPRA1.B4-3D*	Gh_D09G179300.1	D09: 45181994–45182644	651	216	23.64	8.97	chlo: 5, plas: 4, vacu: 2
*GhPRA1.B4-4A*	Gh_A11G031900.1	A11: 2693360–2694010	651	216	23.69	6.73	vacu: 10, plas: 3, extr: 1
*GhPRA1.B4-4D*	Gh_D11G032300.1	D11: 2630603–2631253	651	216	23.69	6.10	vacu: 12, plas: 1, extr: 1
*GhPRA1.C-1A*	Gh_A08G281100.1	A08: 124823067–124823489	423	140	16.10	6.71	plas: 4.5, vacu: 3
*GhPRA1.C-1D*	Gh_D08G271500.1	D08: 67997394–67997933	540	179	20.29	6.06	vacu: 9, golg: 3, plas: 1
*GhPRA1.C-2A*	Gh_A13G220200.1	A13: 104371813–104372518	606	201	22.70	5.11	vacu: 11, plas: 1, extr: 1
*GhPRA1.C-2D*	Gh_D13G223400.1	D13: 59483095–59483754	591	196	22.08	8.05	vacu: 7, plas: 2.5, golg: 2
*GhPRA1.D-2A*	Gh_A09G247600.1	A09: 81208181–81208732	552	183	20.15	4.71	plas: 8, ER: 2, chlo: 1
*GhPRA1.D-2D*	Gh_D09G238600.1	D09: 50472807–50473358	552	183	20.20	4.59	plas: 8, ER: 3, chlo: 1
*GhPRA1.E-1A*	Gh_A11G135200.1	A11: 14400779–14401303	525	174	19.28	7.95	plas: 8, chlo: 3, ER: 3
*GhPRA1.E-1D*	Gh_D11G136100.1	D11: 12800664–12800954	291	96	11.19	10.84	chlo: 7, plas: 6, ER: 1
*GhPRA1.E-2A*	Gh_A07G234800.1	A07: 93082823–93083368	546	181	20.04	9.68	chlo: 7, plas: 6, ER: 1
*GhPRA1.E-2D*	Gh_D07G232500.1	D07: 55224484–55225535	546	181	20.17	9.68	chlo: 7, plas: 6, ER: 1
*GhPRA1.F2-1A*	Gh_A02G142200.1	A02: 80712703–80713260	558	185	20.72	9.09	plas: 10, golg: 2, vacu: 1
*GhPRA1.F2-1D*	Gh_D03G062200.1	D03: 10655558–10656115	558	185	20.76	8.07	plas: 4, vacu: 3, ER: 3
*GhPRA1.F2-2A*	Gh_A10G049300.1	A10: 6090256–6090804	549	182	20.53	9.09	plas: 5, golg: 3, chlo: 2
*GhPRA1.F2-2D*	Gh_D10G062400.1	D10: 6067187–6067735	549	182	20.47	7.93	plas: 7, golg: 3, vacu: 2
*GhPRA1.F2-3D*	Gh_D11G280400.1	D11: 57861036–57861632	597	198	21.96	8.82	plas: 7, ER: 3, chlo: 1
*GhPRA1.G2-1A*	Gh_A08G031500.1	A08: 3124048–3125076	801	266	28.99	8.76	plas: 7, nucl: 2, vacu: 2
*GhPRA1.G2-1D*	Gh_D08G031300.1	D08: 2941239–2941814	576	191	20.78	7.09	plas: 4.5, vacu: 3, ER: 3
*GhPRA1.H-1A*	Gh_A04G042500.1	A04: 7308990–7310582	717	238	26.79	9.14	vacu: 8, plas: 2, extr: 2
*GhPRA1.H-1D*	Gh_D05G353900.1	D05: 56125424–56126730	717	238	26.84	9.30	vacu: 9, plas: 2, extr: 1

## Data Availability

The data analyzed in the current study are included in this article.

## Supplementary Materials

The following are available online at https://www.mdpi.com/article/10.3390/genes13050765/s1, Figure S1: The conserved motifs identified in GhPRA1 proteins; (A) phylogenetic tree of GhPRA1 proteins; (B) the top ten conserved motifs in GhPRA1 proteins; (C) logos of the ten conserved motifs in GhPRA1 proteins, Figure S2: Expression patterns of *GhPRA1* genes by Vd991 infection in RNA-Seq datasets, m, mock; v, Vd991 infection, Figure S3: Expression profiles of *GhPRA1.B1-1A* under Vd991 infection by RT-qPCR, Figure S4: Expression levels of *GhPRA1.B* group members in *GhPRA1.B1-1A* silencing cotton plants, Table S1: Primers used in this study, Table S2: The amino acid sequences and functional annotation of the conserved motifs, Table S3: *Cis*-elements detected in GhPRA1 promoter regions.
