# Supplementary material for: Analysis of the PRA1 Genes in Cotton Identifies the Role of GhPRA1.B1-1A in Verticillium dahliae Resistance
Source: Genes (Basel). 2022 Apr 26;13(5):765. doi: 10.3390/genes13050765 (PMC9141244; doi:10.3390/genes13050765)
Supplement: Supplementary file 1 [file genes-13-00765-s001.zip › Supplementary Figures.pdf]

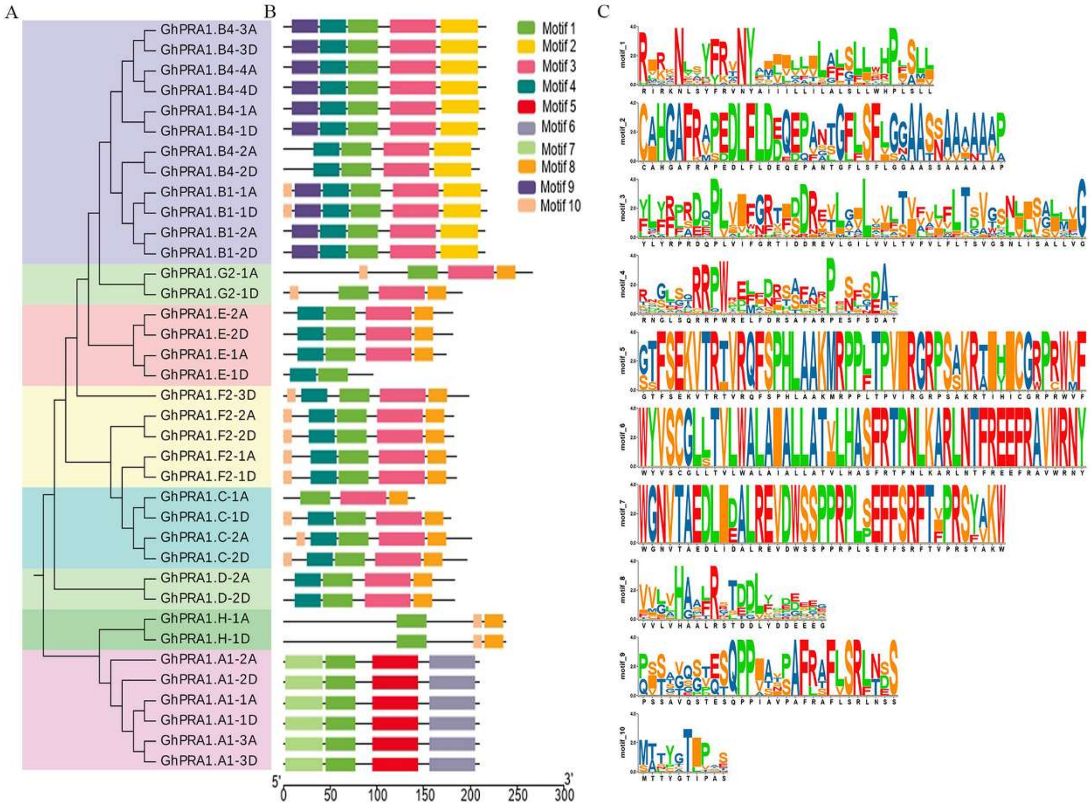

Figure S1: The conserved motifs identified in GhPRA1 proteins. (A) phylogenetic tree of GhPRA1 proteins; (B) the top ten conserved motifs in GhPRA1 proteins; (C) logos of the ten conserved motifs in GhPRA1 proteins.

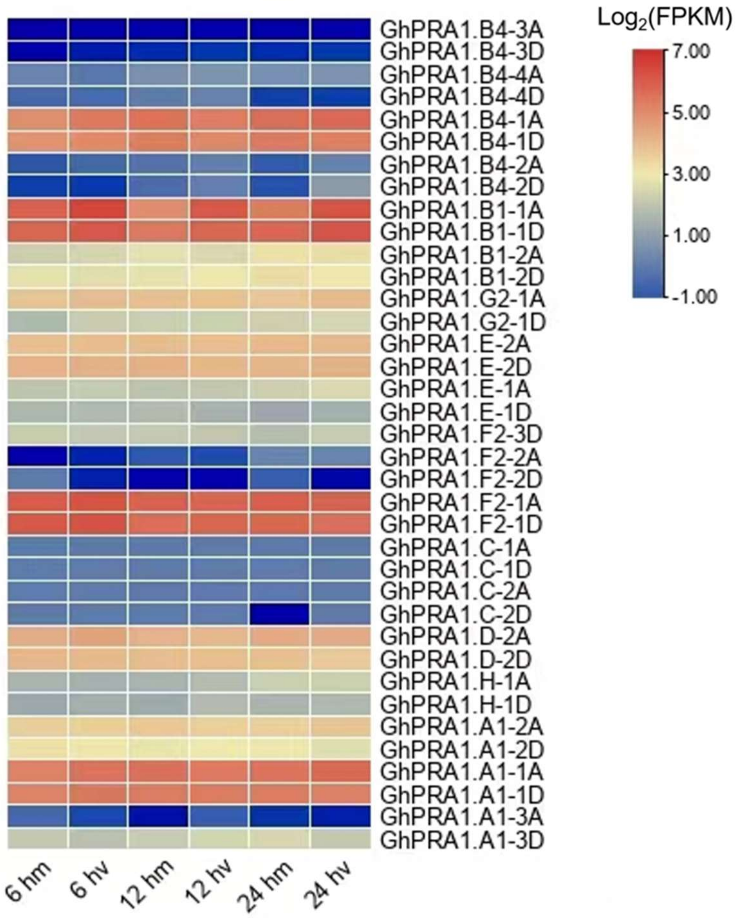

Figure S2: Expression patterns of *GhPRA1* genes by Vd991 infection in RNA-Seq datasets, h, hour; m, mock; v, Vd991 infection.

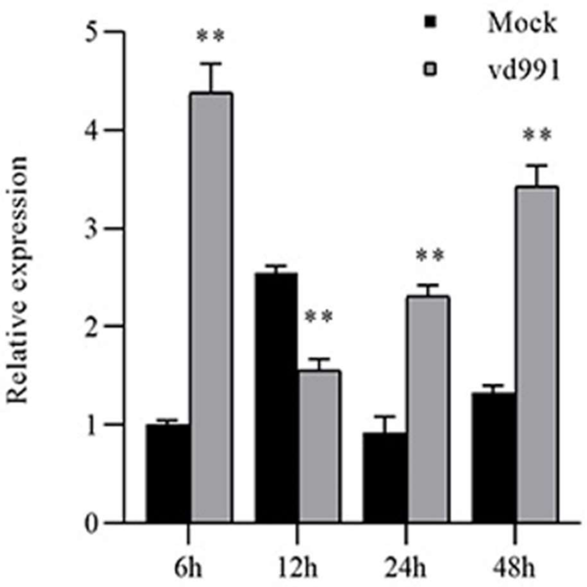

Figure S3: Expression profiles of *GhPRA1.B1-1A* under Vd991 infection by RT-qPCR.

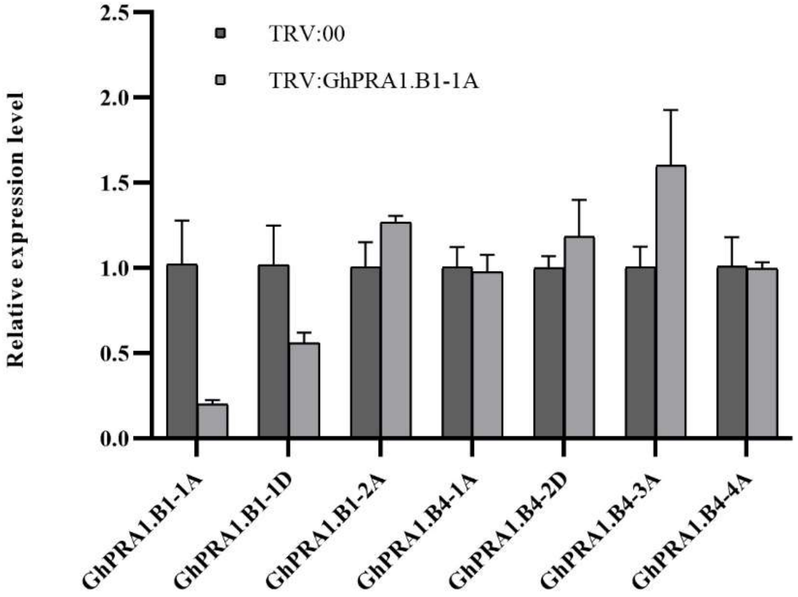

Figure S4: Expression levels of *GhPRA1.B* group members in *GhPRA1.B1-1A* silencing cotton plants.
