# Supplementary material for: Analysis of the PRA1 Genes in Cotton Identifies the Role of GhPRA1.B1-1A in Verticillium dahliae Resistance
Source: Genes (Basel). 2022 Apr 26;13(5):765. doi: 10.3390/genes13050765 (PMC9141244; doi:10.3390/genes13050765)
Supplement: Supplementary file 1 [file genes-13-00765-s001.zip › Table S2.pdf]

Table S2 Amino acid sequences and functional annotation of the conserved motifs.

| Name | Sequences | Annotation |
|---|---|---|
| Motif 1: | RIRKNLSYFRVNYAIIILLILALSLLWHPLSLL | PRA1 family protein |
| Motif 2: | CAHGAFRAPEDLFLDEQEPANTGFLSFLGGAASNAAAA AAP | None |
| Motif 3: | YLYRPRDQPLVIFGRTIDDREVLGILVVLTVFVLFLTDV GSNLJSAJLVG | PRA1 family protein |
| Motif 4: | RNGLSQRRPWKELFBRSAFAKPESFSDAT | PRA1 family protein |
| Motif 5: | GTFSEKVTRTVRQFSPHLAAKMRPPLTPVIRGRPSAKRT IHICGRPRWVF | None |
| Motif 6: | WYVSCGLLTVLWALAIALLATVLHASFRTPNLKARLN TFREEFRAVWRNY | Popeye protein conserved region |
| Motif 7: | WGNVTAEDLIDALREVDWSSPPRPLSEFFSRFTIPRSYA KW | PRA1 family protein |
| Motif 8: | VVLVHAALRRTDDLYDDEEEG | PRA1 family protein |
| Motif 9: | PISAVQSTZSQPPIATPAFRAFLSRLNDS | None |
| Motif 10: | MTTYGTIPAS | None |
